# TIMS Techniques for Measuring Strontium Isotope Ratios in Apatite Grains and Application to Igneous Rock Petrology

**DOI:** 10.1002/rcm.70202

**Published:** 2026-10-25

**Authors:** Carla Neto, Claudio de Morisson Valeriano, Gabriel Paravidini, Henrique Bruno, Manuela Carvalho, Rasec Almeida, Caroline Peixoto, Samuel Bersan, Mariana Carvalho, Monica Heilbron

**Affiliations:** ^1^ PPGG ‐ Programa de Pós‐graduação em Geociências (FGEL‐UERJ) Rio de Janeiro Brazil; ^2^ LAGIR ‐ Laboratório de Geocronologia e Isótopos Radiogênicos (FGEL‐UERJ) Rio de Janeiro Brazil; ^3^ TEKTOS ‐ Grupo de Pesquisa em Geotectônica (FGEL‐UERJ) Rio de Janeiro Brazil; ^4^ FAPERJ – Fundação Carlos Chagas Filho de Amparo à Pesquisa do Estado do Rio de Janeiro Rio de Janeiro Brazil; ^5^ CAp‐UERJ ‐ Departamento de Matemática e Desenho Universidade do Estado do Rio de Janeiro (UERJ) Rio de Janeiro Brazil

**Keywords:** apatite, ion exchange chromatography, radiogenic isotopes, strontium isotopes, thermal ionization mass spectrometry

## Abstract

**Rationale:**

This study evaluates the potential of Sr isotopic compositions in apatite grains from magmatic rocks as a proxy for initial ^87^Sr/^86^Sr ratios, addressing the need for alternative approaches to conventional whole‐rock analyses in isotopic studies of granitoid systems.

**Methods:**

Chemical and spectrometric procedures were optimized and validated for the determination of Sr isotopic ratios in apatite by TIMS. Three representative samples from the Ribeira Orogen, Brazil, were selected, and apatite Sr isotopic compositions were compared with initial whole‐rock ^87^Sr/^86^Sr ratios calculated from U–Pb zircon crystallization ages, whole‐rock isotopic ratios, and Rb and Sr concentrations. The reliability of the analytical approach was evaluated using the EN‐1 and Durango apatite reference materials.

**Results:**

Apatite grains yielded Sr concentrations ranging from 80 to 950 μg g^−1^, with total Sr masses between 0.5 and 50 ng. Comparisons between apatite‐derived and whole‐rock initial ^87^Sr/^86^Sr ratios show isotopic variability below 0.05%, indicating high analytical precision. Validation results demonstrate consistent agreement between methods, with uncertainties within 0.5%.

**Conclusions:**

The proposed TIMS‐based method for Sr isotope analysis in apatite grains provides a potential approach for investigating the initial Sr isotopic compositions of granitoid magmatic systems. This approach represents a cost‐effective and time‐efficient alternative to traditional whole‐rock analyses, with strong potential for large‐scale isotopic applications in orogenic terrains.

## 1 Introduction

The use of apatite to solve several complex geological problems has grown remarkably in recent years, becoming a promising tool for addressing fundamental questions in Earth's history [1, 2]. Apatite is a calcium phosphate [Ca_5_(PO_4_)_3_ (F, Cl, OH)] widely distributed in the lithosphere and is present in most rock types, environments, and geological processes [3, 4, 5, 6, 7, 8]. Particularly, in studies of orogens, which are tectonically complex regions, the application of Sr initial ratios in petrogenetic studies with regional scope offers a valuable tool to differentiate mantellic and crustal sources, offering opportunities for the interpretation of processes such as partial melting, crustal contamination, and mantle–crust interaction [9, 10, 11, 12]. The very low Rb/Sr ratios in apatite make it particularly suitable for obtaining the initial ^87^Sr/^86^Sr ratio of rocks, which has been widely used to investigate their origin [13, 14, 15, 16, 17, 18, 19, 20, 21, 22, 23, 24, 25]. Consequently, apatite has been widely used as a proxy for estimating the initial ^87^Sr/^86^Sr ratio of igneous rocks.

Given the relevance of Sr isotopic ratios in apatite for petrogenetic applications, it is essential to optimize accessible and viable techniques for obtaining such data. Although both apatite analysis and the use of Sr isotopes in apatite as a proxy for whole‐rock isotopic compositions are already well established in the literature cited above and are predominantly applied using in situ techniques such as LA‐MC‐ICP‐MS, previous studies have generally emphasized geological applications, whereas detailed analytical workflows, systematic evaluation of analytical reproducibility, and direct comparisons between Sr isotopic compositions obtained in apatite and calculated whole‐rock initial ratios are only briefly discussed or treated as secondary aspects.

Traditionally, high‐precision Sr isotopic analyses are performed using TIMS and MC‐ICP‐MS, which represent the state of the art due to their high accuracy and excellent reproducibility. TIMS remains the reference method because of its high ionization efficiency and minimal matrix effects, enabling highly precise and robust isotopic measurements. In contrast, LA‐ICP‐MS has gained prominence by enabling in situ analyses with high spatial resolution and lower sample consumption. However, this technique still faces limitations related to laser‐induced fractionation, mass bias, and isobaric interferences. In addition, its accuracy strongly depends on the availability of well‐characterized reference materials. In this context, TIMS continues to serve a critical role as a standard for validation and the generation of high‐precision isotopic data. Although TIMS provides high‐precision Sr isotope measurements, its application to individual apatite grains presents specific challenges related to the very small amounts of available Sr, which can range from sub‐ng to ng levels. These low‐Sr amounts require efficient dissolution and Sr purification while minimizing procedural blanks and Sr losses. In addition, the separation of Sr from Rb and other matrix elements is important because residual Rb may interfere with ^87^Sr measurements. The conventional chemical procedures developed for larger samples may also involve relatively large resin and reagent volumes and longer preparation times, making their direct application to individual apatite grains less practical. Thus, both approaches constitute the current state of the art in Sr isotope geochemistry [26, 27].

This study aims to optimize and validate a TIMS analytical workflow for Sr isotope determination in apatite using sub‐ng to ng quantities of Sr, through its application to well‐characterized reference materials and apatite grains separated from rocks with variable Sr concentrations. This includes chemical procedures and isotopic measurements, together with a systematic assessment of analytical performance and geological applicability, while also evaluating the potential of apatite as an isotopic proxy for petrogenetic investigations in granitoid systems.

## 2 Materials and Methods

### 2.1 Study Area and Samples

The studied granitoid samples were collected in the Ribeira Belt, a major Neoproterozoic orogenic system that forms part of the Mantiqueira Province in southeastern Brazil ([28, 29, 30] and references therein). This orogenic system comprises a complex array of Neoproterozoic terranes that were diachronically amalgamated between ca. 840 and 520 Ma during the assembly of the West Gondwana supercontinent [31, 32, 33].

Three granitoid samples representing distinct magmatic stages of the orogeny were selected for Sr isotopic analysis of apatite: a precollisional granodioritic gneiss (SMM‐CMM‐172), a syn‐collisional orthogneiss (BG‐200), and a postcollisional granitoid (BG‐14B). These samples were selected because whole‐rock Rb and Sr concentrations, Nd and Sr isotopic data, and zircon U–Pb crystallization ages are available [34, 35].

A summary of the geological setting, lithological classification, whole‐rock geochemical data, isotopic Nd‐Sr and geochronological constraints relevant to this study is provided in Table 1. Sample descriptions, petrographic characteristics, and representative thin‐section photomicrographs are available in Figure S1 and Text S1. Although these geological characteristics do not directly influence the TIMS analytical procedure, they are important for interpreting the isotopic results and evaluating the relationship between apatite and host‐rock Sr isotope compositions. In addition to documenting the origin and composition of the samples, the whole‐rock Rb and Sr concentrations and the crystallization ages are required for calculating the initial ^87^Sr/^86^Sr ratios. A detailed description of the age‐corrected ^87^Sr/^86^Sr calculations and the propagated uncertainties for each calculated initial isotopic ratio is provided in Equation S1.

**TABLE 1:** Summary of the samples analyzed in this study.

Sample	Stage collisional	Lithology	Geological unit	Coordinates (WGS84)	Age (Ma) (±2 s)	Rb (μg g−^1^) (±2 s)	Sr (μg g−^1^) (±2 s)	ɛ Nd (i)	^87^Sr/^86^Sr_(measured)_ ± (2 s)
SMM‐CMM‐172	Pre—	Granodioritic gneiss	Rio Negro Complex	−21.75413, −42.19951	629 ± 10	128 ± 1.3	287 ± 2.9	−8.4	0.722254 ± 6
BG‐200	Syn—	Orthogneiss	Bingen Unit	−22.55499, −43.22531	602.2 ± 4.9	137 ± 1.4	321 ± 3.2	−9.8	0.724903 ± 7
BG‐14B	Post—	Granitoid	Rio Negro Complex	−22.603914, −43.28459	518.3 ± 1.9	97 ± 1.0	221 ± 2.2	−7.2	0.720435 ± 11

*Note:* Whole‐rock Rb and Sr concentrations, whole‐rock Nd and Sr isotopic data, and zircon U–Pb crystallization ages were used to calculate the initial ^87^Sr/^86^Sr ratios discussed in this study. Data for sample SMM‐CMM‐172 are from Peixoto et al. [35], whereas data for all other samples are from Neto [34].

### 2.2 Analytical Procedures in Apatite Grains

#### 2.2.1 ^87^Sr/^86^Sr Measurements by TIMS

All chemical and mass spectrometric procedures related to Sr isotopic analyses were conducted at the Laboratory of Geochronology and Radiogenic Isotopes (LAGIR), Rio de Janeiro State University (UERJ), in clean room facilities equipped with HEPA filtration and maintained under positive pressure.

The analytical procedure was optimized to enable precise Sr isotopic measurements in apatite grains containing sub‐ng to ng quantities of Sr. The optimization focused on simplifying the grain digestion and chromatographic separation by reducing resin and reagent volumes and time of dissolution when compared with conventional whole‐rock Sr extraction procedures reported by Neto et al. [36].

The chemical and analytical procedures employed in this study largely follow the approach described by Charlier et al. [37]; however, specific modifications were introduced, from the apatite digestion step, considering that Charlier et al. [37] worked with plagioclase, to the separation procedures, including the type of column and the reagents used for column and resin cleaning. Our contribution therefore consists of developing an analytical workflow specifically adapted to the determination of Sr isotopic ratios by TIMS in individual apatite grains containing very small amounts of Sr, on the order of subnanogram to nanogram quantities. The performance of the analytical procedure for the determination of Sr isotopic ratios in apatite was evaluated using the EN‐1 and Durango Apatite reference materials and was subsequently applied to analyses of individual and multiple apatite grains from granitoid samples with different Sr concentrations.

Before dissolution, apatite grains were leached in 0.5‐M acetic acid at 90°C for 1 h 30 min to remove possible surface contaminants [38]. The grains were subsequently rinsed three times with ultrapure H_2_O and dried at room temperature. Single‐grain and multigrain apatite separates were digested in 200 μL of ultrapure HNO₃ (65%) at 140°C for 24 h [39]. The EN‐1 reference material was dissolved in 1 M HCl at 90°C for 24 h.

After digestion, the same apatite aliquots used for Sr mass determination were employed for Sr isotope ratio measurements by TIMS, ensuring direct correspondence between the estimated Sr mass and isotopic data obtained for each sample.

Strontium was separated from the sample matrix using Sr‐Spec resin (50–100 μm; Eichrom Technologies) packed in microcolumns (3.1‐mm internal diameter, 10 mm length) containing approximately 70 μL of resin [37]. After complete evaporation, samples were redissolved in 200 μL of 3 M HNO₃ and loaded onto the column. Matrix elements were removed by four successive washes with 3 M HNO₃, using 50, 100, 150, and 200 μL in each successive fraction, respectively. Strontium was subsequently eluted with 0.05 M HNO₃, collected in three successive 100‐μL fractions, for a total elution volume of 300 μL. The separation procedure followed Charlier et al. [37] (Table S1).

The chromatographic procedure was calibrated using a Durango apatite Reference Material (RM) [24, 40, 41] grain containing approximately 20 ng of Sr. Successive elution fractions were analyzed by TIMS, and an elution curve was constructed by plotting the Sr and Rb signal intensity (counts per second [cps]) versus aliquot volume (Figure S2). For lower intensity signals, measurements were performed using the secondary electron multiplier (SEM) and recorded in cps, whereas higher signals were measured using Faraday cups. A conversion factor of 1 mV = 62 500 cps was used to compare signals obtained with the two detection systems. This approach allowed identification of the Sr elution interval free from Rb interference. Based on the elution profile, the Sr collection window adopted for all samples corresponded to the 300‐μL fractions of 0.05 M HNO₃.

The Sr amount obtained for each sample through chemical analysis and the estimated masses were used to determine the masses of Sr loaded onto the column for isotopic analyses. The Sr concentrations in apatite range from approximately 80 to 950 μg g^−1^, yielding Sr masses of 0.5 to 5 ng for single grains and up to ~50 ng for multigrains, covering mass variations of up to three orders of magnitude.

After evaporation, the purified Sr fraction was loaded onto single Re filaments together with 1 μL of TaF₅ activator solution, following Charlier et al. [37]. This activator enhances Sr ionization efficiency during TIMS analysis and promotes stable ion beam generation, particularly for samples containing very small amounts of Sr, thereby improving the analytical performance of low‐Sr apatite separates. The best ionization temperature for Sr analyses in Re filaments varies from 1350°C to 1450°C [1]. Isotopic measurements were performed on a Triton TIMS using 100 acquisition cycles, an integration time of 4.194 s, and an idle time of 3 s. In addition to the four masses of the Sr isotopes, the ^85^Rb mass was monitored and measured to correct for any possible interference with ^87^Sr. Exponential law mass fractionation correction of the ^87^Sr/^86^Sr isotope ratio was based on the ^88^Sr/^86^Sr ratio of 8.375209 [42]. Correction of possible isobaric interference was based on the ^87^Rb/^85^Rb ratio of 0.386 [43, 44]. The measured ^87^Sr/^86^Sr ratios were normalized through repeated analyses of the NIST SRM 987 Sr isotopic reference material, which was used to monitor and correct instrumental bias during TIMS measurements. The ^87^Sr/^86^Sr ratio result from NIST SRM 987 was 0.710238 ± 0.000045 (2 s, *n* = 32), which is close to the recommended value of ^87^Sr/^86^Sr = 0.710248 ± 0.000008. The operating parameters and analytical conditions used for Sr isotopic measurements by TIMS are summarized in Table S2.

The accuracy of the Sr isotope measuments on TIMS was assessed through Sr isotopic analyses of EN‐1 (Enewetak lagoon; bivalve shells; [45]) and Durango Apatite, the latter matrix‐compatible with the samples studied. These RMs were used to check external reproducibility and to empirically validate blank conditions. The independent Student's test and one‐way ANOVA were used to assess significant differences between two or more groups from the average. To verify the reliability assumptions of the tests, the normality test of data distribution [46] and the homogeneity of variances test [47] were used, both yielded by *p*‐values > 0.05, showing groups with normal distribution and homogeneous variances. All statistical parameters were obtained using Jamovi 1.2.27 [48, 49].

#### 2.2.2 ICP‐MS Analysis of Sr in Apatite

Mass determination of Rb and Sr fractions in apatite grains was performed using the ICP‐MS Element XR (ThermoScientific) at the Isotopic Geology Laboratory (LAGIS) of the Institute of Geosciences of the State University of Campinas (Unicamp). To ensure the quality of the measurements, the certified whole‐rock reference material BRP‐1 (basalt) was analyzed, obtaining results consistent with the uncertainties associated with RM, as described by Cotta and Enzweiler [50]. The accuracy was evidenced by an average variation coefficient of 1.4% in duplicate samples. Furthermore, the blank samples presented concentrations below the detection limit for all the elements analyzed.

## 3 Results

### 3.1 Measured Isotope Compositions of EN‐1 and Durango Apatite

The results of EN‐1 and Durango Apatite analyses are presented in Tables S3 and S4, which include individual measurements, statistical parameters, and reference values. The average of ^87^Sr/^86^Sr measured in EN‐1 is 0.709174 ± 0.000032 (2 s, *n* = 15), with a relative standard deviation (RSD) of 0.002% (Table S3). The amounts of Sr loaded in the column ranged from 1 to 30 ng. Repeated analyses of the ^87^Sr/^86^Sr ratio from EN‐1 performed at LAGIR‐UERJ are compared with values published in the GeoReM database (http://georem.mpch‐mainz.gwdg.de) that provide an average of 0.709172 ± 0.000027 (2 s, *n* = 24; see Table S5). The reproducibility of the value measured at LAGIR‐UERJ, which is less than 0.0003%, is comparable to values reported in GeoReM (Table S3).

Sr isotopic analyses of the Durango Apatite (486 μg g^−1^; [24]) were performed on apatite grain fragments. The analyzed apatite aliquots ranged from 1 to 70 μg in sample mass, corresponding to approximately 0.5 to 35 ng of Sr after chemical digestion, depending on the Sr concentration of each sample. These analyses yielded an average ^87^Sr/^86^Sr ratio of 0.706334 ± 0.000023 (2 s, *n* = 16), with RSD of 0.002% (Table S4). The difference between the value reported by Yang et al. [24] for the ^87^Sr/^86^Sr ratio (0.706328 ± 0.000023) by LA‐ICP‐MS and Horstwood et al. [40] for the ^87^Sr/^86^Sr ratio (0.706327) by TIMS and the value obtained in this study is less than 0.001%, demonstrating the consistency and reproducibility of the analytical data (Table S4).

### 3.2 Whole‐Rock and Apatite ^87^Sr/^86^Sr Ratios of Granitoid Samples

The hand‐picked apatite grains were carefully selected to ensure they belonged to the same population, based on their morphology, color, and size, using a magnifying glass. Typically, these grains exhibit proportions of 2:1 (c‐axis ~150 μm) to 3:1 (c‐axis ~200 μm), corresponding to masses of approximately 3 μg. All raw data on the isotopic composition of the apatite measurements by TIMS are provided in Table S6 and Table S7.

**SMM‐CMM‐172**: The whole‐rock isotopic analysis provides an initial εNd of −8.4 [35] and an initial ^87^Sr/^86^Sr ratio of 0.710833 ± 0.000628 (U, *k* = 2). The apatite ^87^Sr/^86^Sr ratios range from 0.713948 to 0.714727, with an average isotopic composition of 0.714313 ± 0.000442 (2 s, *n* = 17), RSD of 0.03%, and an apatite Sr content of 945 μg g^−1^.

**BG‐200:** The whole‐rock isotopic analysis indicates an initial εNd of −9.8 [34] and initial ^87^Sr/^86^Sr ratio of 0.714438 ± 0.000498 (U, *k* = 2). The apatite ^87^Sr/^86^Sr ratios vary between 0.715920 and 0.717079, with an average isotopic composition of 0.716527 ± 0.000653 (2 s, *n* = 14), RSD of 0.05%, and [Sr_ap_] of 233 μg g^−1^.

**BG‐14B:** The whole‐rock isotopic data provide an initial εNd of −7.2 [34] and initial ^87^Sr/^86^Sr ratios of 0.711182 ± 0.000420 (U, *k* = 2). The average of the ^87^Sr/^86^Sr isotopic ratios measured in apatite grains in sample BG‐14B is 0.712937 ± 0.000344 (2 s, *n* = 12), ranging from 0.712656 to 0.713222, with RSD of 0.02% and apatite Sr content of 83 μg g^−1^.

## 4 Discussion

### 4.1 Relationship Between ^87^Sr/^86^Sr Isotopic Fractionation and the Sr Contents in the Reference Materials and Samples

To evaluate possible significant isotopic fractionations of the ^87^Sr/^86^Sr ratios analyzed in the reference materials, based on the amount of Sr (ng) loaded in the chromatographic column in different orders of magnitude (10^1^, 10^0^, and/or 10^−1^), groups were formed with the individual values measured for comparison of the averages that will be described below. The difference among the mean values obtained for the different analytical groups of each reference material was lower than 0.0003%. The excellent reproducibility achieved for the EN‐1 and Durango apatite reference materials, which were used throughout the development and validation of the analytical procedure presented in this study, demonstrates the robustness of the method and supports the reliability of the isotopic results obtained (Figure 1).

**FIGURE 1:**
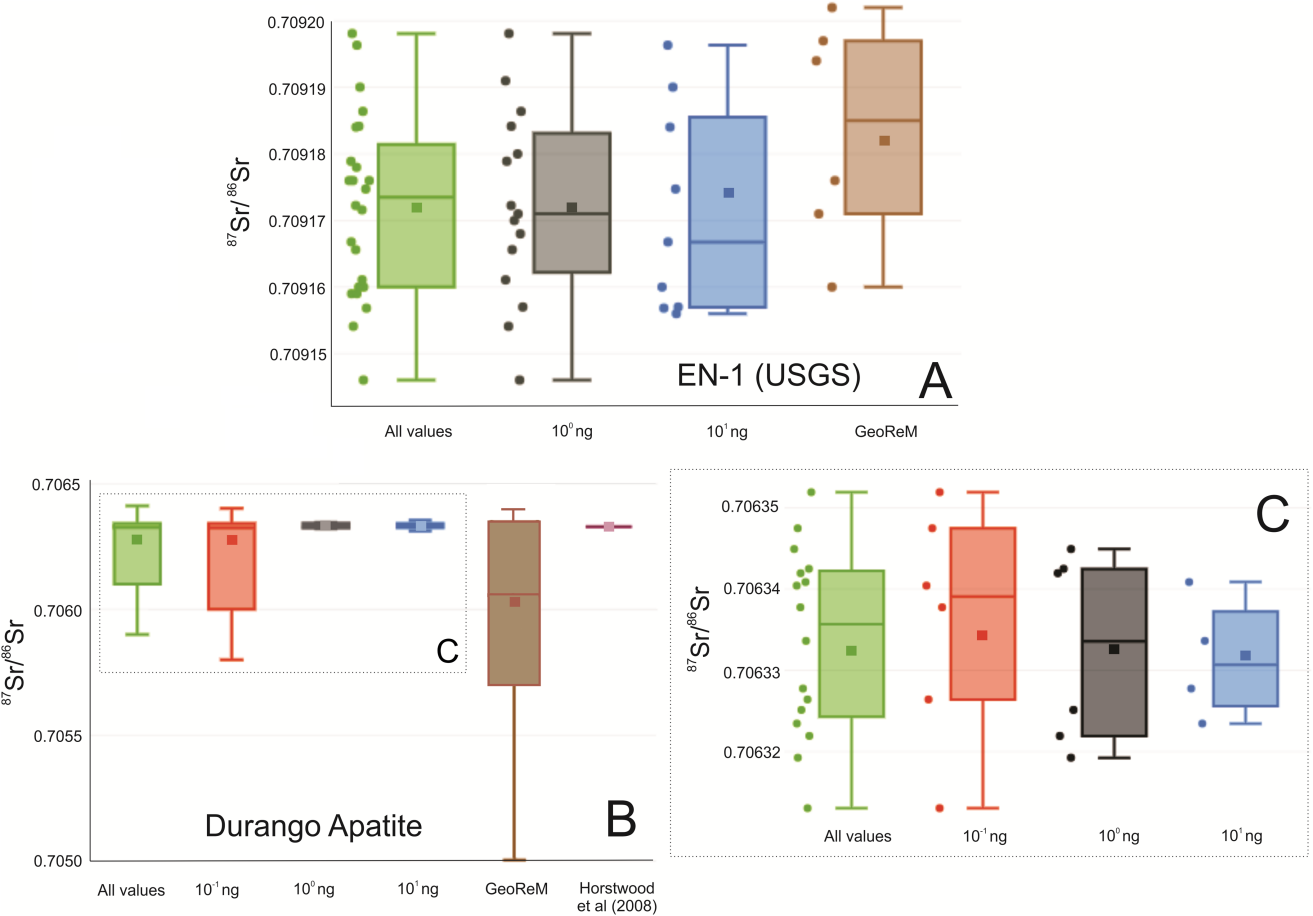
Representation of the measured ^87^Sr/^86^Sr isotopic ratios for the EN‐1 and Durango Apatite reference materials as boxplots and comparison of groups separated according to the order of magnitude of the Sr mass loaded onto the column. (A) EN‐1, (B) Durango Apatite, and (C) expansion of the dashed rectangle shown in (B). The green circles and boxplot represent all ^87^Sr/^86^Sr ratios measured at LAGIR‐UERJ (“LAGIR all values” group). The horizontal line within each box represents the median, whereas the vertical lines indicate the interquartile range. The “LAGIR all values” group encompasses all individual ^87^Sr/^86^Sr ratios obtained for each reference material, whereas the other groups are subsets of this dataset, comprising values corresponding to different orders of magnitude of Sr mass loaded onto the column: 10^−1^ ng (red), 10^0^ ng (gray), and 10^1^ ng (blue). The brown boxplot represents the GeoReM data, and the orange horizontal bar corresponds to the only published ^87^Sr/^86^Sr value for Durango Apatite obtained by TIMS and reported in the literature.

The absence of statistically significant differences among the different Sr mass groups for both EN‐1 and Durango Apatite demonstrates that the proposed TIMS procedure provides stable and reproducible ^87^Sr/^86^Sr ratios over a wide range of Sr loads. Although an apparent trend of increasing ^87^Sr/^86^Sr values with decreasing Sr mass can be observed, particularly for Durango apatite, the magnitude of this variation is small relative to the analytical dispersion. When analyzing samples with very low‐Sr masses, greater variability in ion beam intensity and signal stability can contribute to the scatter of individual measurements, whereas higher Sr loads generally yield more stable ion beam signals. However, within the range investigated in this study, these effects did not result in statistically significant differences in the measured ^87^Sr/^86^Sr ratios. The agreement between the measured values and previously published reference data further confirms the analytical accuracy of the method and indicates that no significant isotopic fractionation was produced during chemical separation or TIMS measurements. These results demonstrate that the analytical protocol remains robust even under low‐Sr conditions, which represent one of the principal analytical limitations in apatite isotope studies.

The comparison between single‐grain and multigrain analyses for samples SMM‐CMM‐172, BG‐200, and BG‐14B (Figure 2) also supports the isotopic homogeneity of the analyzed apatite populations. The close agreement among the average ^87^Sr/^86^Sr ratios obtained from single‐grain and multigrain analyses indicates that natural variations in Sr concentration among the analyzed apatite grains did not result in a systematic difference in the measured Sr isotope ratios under the analytical conditions adopted in this study. Furthermore, the reproducibility obtained for analyses involving Sr masses between 0.5 and 50 ng demonstrates that the method maintains consistent analytical performance even for apatite grains with very low=Sr contents.

**FIGURE 2:**
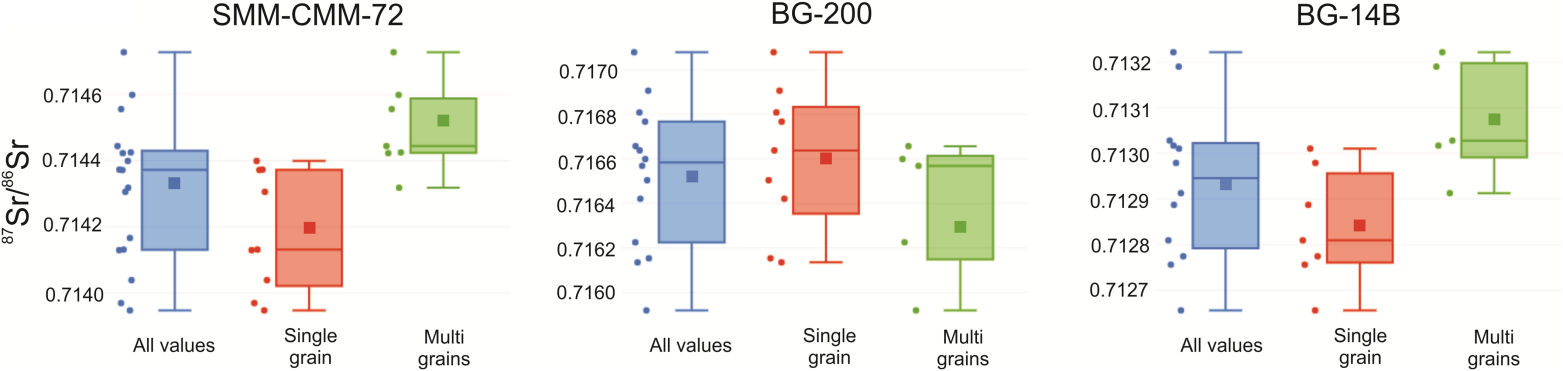
Representation of the measured ^87^Sr/^86^Sr isotopic ratios for the samples as boxplots: SMM‐CMM‐172, BG‐200, and BG‐14, together with the corresponding GeoReM values (Table S8) and the TIMS value reported by Horstwood et al. [40]. The blue circle represents the individual values of the “All values” group (complete set of isotopic measurements). The red and green circles correspond to the values of the “Single‐grain” and “Multigrain” subgroups (derived from the complete set), respectively. The horizontal line within each box represents the median, whereas the vertical lines indicate the interquartile range.

Although direct chemical analyses are not essential for the application of the proposed technique, preliminary estimates of apatite Sr concentrations may provide a useful approach for evaluating the expected analytical performance before isotopic measurements. Such estimates can be based on whole‐rock geochemistry, petrographic classification, tectonic setting, or empirical relationships previously proposed for granitoid apatites [13, 51, 52]. In general, apatite Sr concentrations tend to follow the range 0.25 ≤ Sr_apatite_/Sr_whole‐rock_ ≤ 1 [13], allowing a preliminary assessment of the expected Sr load during isotopic analysis.

To provide practical guidance for future applications of the method, Table 2 summarizes the relationships among rock type, SiO_2_ content, whole‐rock Sr concentration, apatite Sr concentration, and the minimum number of apatite grains required for TIMS analyses in the studied samples. Apatite Sr concentrations range from 83 to 945 μg g^−1^, resulting in substantial differences in the amount of material required for isotopic analysis. Although the limited number of samples does not allow the establishment of a statistically robust predictive relationship, the data suggest that apatite Sr concentrations are influenced by the degree of magmatic differentiation, as reflected by whole‐rock SiO_2_ contents. Because SiO_2_ content and rock classification are commonly available parameters, they may serve as practical first‐order proxies for estimating apatite Sr concentrations, and, consequently, the number of grains required for TIMS isotopic analyses. Future studies involving a larger number of samples are needed to further evaluate and quantify this relationship.

**TABLE 2:** Relationship between rock composition, whole‐rock Sr concentration, apatite Sr concentration, and the approximate number of apatite grains required for TIMS Sr isotope analyses.

Sample	Rock type	SiO_2_ (wt%)	Whole‐rock Sr (μg g−^1^)	Apatite Sr (μg g−^1^)	Approximate Sr per apatite grain (ng)	Suggested number of grains (Estimated assuming na apatite grain mass of ~3 μg)
SMM‐CMM‐172	Granodiorite	64.8	287	945	~2.8–3.0	1
BG‐200	Granite	69.7	321	233	~0.7	3–5
BG‐14B	Diorite	56.2	221	83	~0.25–0.30	8–10

### 4.2 Comparison of Initial ^87^Sr/^86^Sr Ratios Obtained From Apatite Grains and From Whole‐Rock Analysis

Replicate analyses of apatite grains from samples SMM‐CMM‐172, BG‐200, and BG‐14B yielded consistent ^87^Sr/^86^Sr ratios, with RSDs lower than 0.05% (Figure 3). These results indicate that the TIMS analytical procedure developed in this study provides reproducible Sr isotopic measurements for apatite separates from igneous rocks.

**FIGURE 3:**
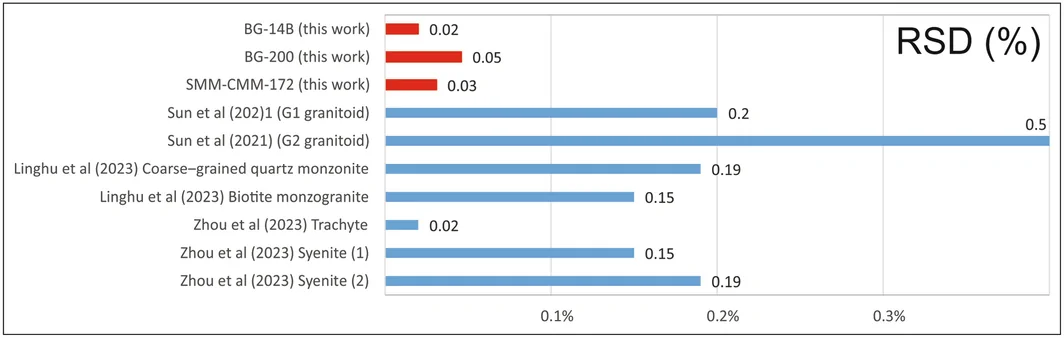
Comparison of the RSDs (relative standard deviations) of the 87Sr/86Sr ratios of the apatite grains from this study with those from literature, reflecting the geological heterogeneity of the natural grains.

Previous studies have reported variations in ^87^Sr/^86^Sr ratios among apatite grains from granitoid and alkaline rocks, reflecting geological heterogeneity at the grain scale (e.g., [53, 54, 55]; Figure 3). Because these studies employed in situ LA‐MC‐ICP‐MS analyses, a direct comparison of analytical uncertainties with the TIMS results presented here is not appropriate. Therefore, the literature data are used only to provide geological context regarding the range of Sr isotopic compositions reported for apatite in igneous systems.

The variability observed among replicate apatite analyses in this study demonstrates that the developed TIMS methodology yields reproducible ^87^Sr/^86^Sr ratios from small apatite aliquots, supporting its application in regional‐scale petrogenetic investigations when compared to whole‐rock isotopic compositions.

The relative percentage difference (RPD) between the calculated initial ^87^Sr/^86^Sr_(WR)_ ratio and the average ratio in the apatite grains is 0.5%, 0.3%, and 0.2% for the SMM‐CMM‐172, BG‐200, and BG‐14B samples, respectively. The regional Sr isotopic fields shown in Figure 4 were defined using published initial ^87^Sr/^86^Sr ratios for pre‐, syn‐, and postcollisional granitoid rocks of the Ribeira Belt. The limits of each field encompass the range of isotopic values reported in previous studies and represent the characteristic isotopic variability of these magmatic suites [30, 35, 56, 57, 58]. Figure 4 and Table 3 present the individual ^87^Sr/^86^Sr ratios obtained in apatite grains, as well as the initial ^87^Sr/^86^Sr ratios calculated from the whole‐rock analyses for each of the studied samples in combination with the initial εNd values, assuming that the initial εNd from whole rock is equivalent to that of the apatite grains, based on Linghu et al. [53]. Figure 4 also shows the relationship between εNd_(t)_ and ^87^Sr/^86^Sr_(t)_ and reinforces the agreement by demonstrating that the Sr isotopic data of the apatite grains and the whole rocks are within the regional fields of the samples SMM‐CMM‐172, BG‐200 and BG‐14B previously defined for granitoid rocks associated with the pre‐, syn‐ and postcollisional Ribeira Orogeny, respectively. It should be noted that the εNd_(t)_ values shown in Figure 4 were compiled from published whole‐rock datasets and are included only as a reference framework to contextualize the Sr–Nd isotopic space. It is important to emphasize that they are not derived from measurements performed on the apatite analyzed in this study. The interpretation presented is based exclusively on variations along the horizontal Sr isotopic axis, which reflects the initial ^87^Sr/^86^Sr ratios obtained from apatite analyses. Therefore, any potential isotopic heterogeneity at the grain scale would not affect the main conclusions of this work, which are focused on the Sr isotopic behavior of apatite relative to the host magmatic system.

**FIGURE 4:**
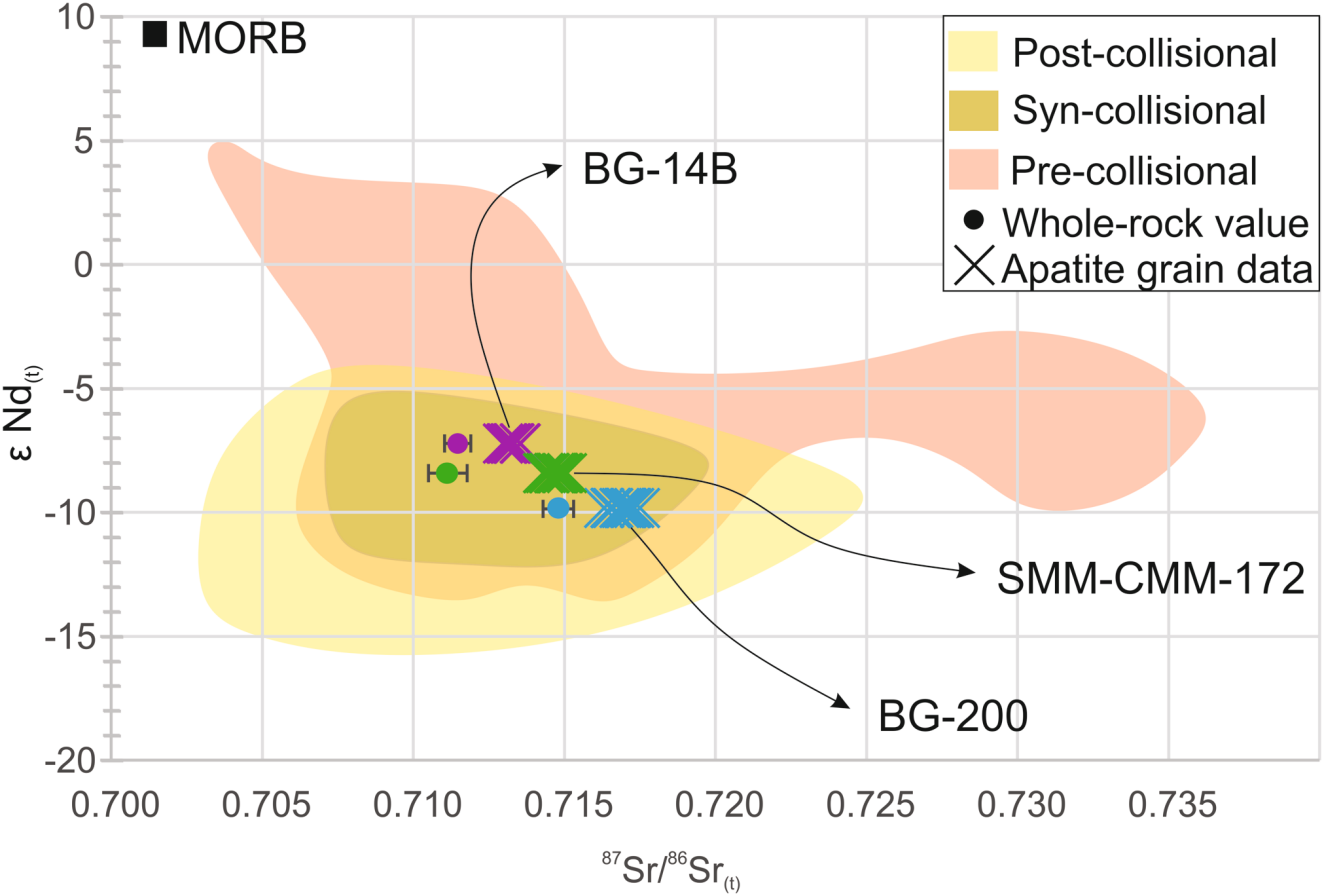
εNd versus ^87^Sr/^86^Sr _(t)_ diagram, assuming equivalent εNd values for whole‐rock samples and apatite grains. The term ^87^Sr/^86^Sr _(t)_ represents the initial strontium isotopic ratio calculated at time *t*, corresponding to the crystallization age of the rock as determined by U–Pb zircon geochronology. Data in plotted fields for pre‐, syn‐, and postcollisional granitoid rocks are from Medeiros et al. [56]; Silva [57]; Machado et al. [59]; Peixoto et al. [35]; Heilbron et al. [30]; Potratz et al. [58].

**TABLE 3:** Summary of the isotopic compositions of the granitoid samples, including initial Sr isotopic ratios calculated from whole‐rock (WR) data, mean values obtained for apatite grains analyzed by TIMS, and their associated uncertainties.

Sample	^87^Sr/^86^Sr_(WR calculated initial)_	U _(k = 2)_	^87^Sr/^86^Sr _(apatite grains)_	2 s	RPD (%)
SMM‐CMM‐172	0.710833	0.000628	0.714313	0.000442	0.5
BG‐200	0.714438	0.000498	0.716527	0.000653	0.3
BG‐14B	0.711182	0.000420	0.712937	0.000344	0.2

*Note:* U—expanded uncertainty associated with the calculated initial ^87^Sr/^86^Sr ratio. Relative percentage difference (RPD) between the calculated initial ratio and the average ratio in the apatite grains.

These results confirm the robustness of the proposed analytical technique and indicate that apatite grains reliably preserve, in some way, the initial Sr isotopic signature of the magmatic systems, even under high‐grade metamorphic conditions. Because apatite has low Rb/Sr ratios and is less susceptible to postmagmatic isotopic re‐equilibration, the ^87^Sr/^86^Sr ratios measured in these grains approximate the original isotopic composition of the magma at the time of crystallization. Consequently, the agreement between apatite‐derived and whole‐rock initial ratios allows the reconstruction of source characteristics and magmatic processes, such as mantle versus crustal contributions, magma mixing, and crustal contamination. In this context, apatite‐based Sr isotopic data provide a viable and efficient tool for tracing the isotopic evolution of granitoid systems and strengthen their application in regional geological reconstruction studies.

### 4.3 Advantages and Limitations of the Technique

The technique developed for the analysis of ^87^Sr/^86^Sr isotopic ratios in apatite grains presents advantages that make it a viable alternative tool for large‐scale studies in different geological contexts when compared to the traditional whole‐rock Sr extraction method reported by Gioia and Pimentel [60] and Neto et al. [36]. The optimized protocol significantly reduces reagent consumption, sample preparation time, and potential procedural blank contributions while maintaining high analytical precision and reproducibility for samples containing low amounts of Sr. The use of smaller columns and reduced reagent volumes was designed to minimize contamination throughout the chemical procedure and is expected to yield low procedural blank levels. This contrasts with conventional whole‐rock dissolution procedures, which typically require larger acid volumes and prolonged digestion times. Strontium chromatographic separation was also optimized for low‐mass samples using reduced resin volumes and shorter elution schemes, thereby increasing analytical throughput and reducing overall processing time (Table 4 and Figure S3).

**TABLE 4:** Comparison between conventional whole‐rock analytical workflows and the optimized apatite TIMS procedure presented in this study.

Parameter	Conventional WR method	Proposed apatite method
Sample mass	~20–100 mg	~3 μg
Acid volume	High	Low
Digestion time	Long (~5 days)	Short (24 h)
Resin volume	High (~4000 μL)	Reduced (~70 μL)
Sr load	μg‐level	ng‐level
Analytical throughput	Lower	Higher
Sample preparation time	Longer (5 h)	Reduced (2 h30)
Number of purification steps	Multiple	Reduced

*Note:* Conventional WR method Gioia and Pimentel [59] and Neto et al. [36].

Among its main advantages are the reduction of analytical costs and time, without compromising the accuracy of the results. Conventional whole‐rock approaches generally require larger sample volumes, longer preparation times, and greater reagent consumption, limiting their application in studies involving scarce material or mineral‐scale isotopic investigations. In contrast, the proposed method enables reliable analyses of samples containing as little as 0.5 ng of Sr. In addition, the proposed procedure enables precise and reproducible TIMS measurements using sub‐ng to ng quantities of Sr, while substantially reducing analytical costs, reagent consumption, and overall processing time. The optimized use of reagents, smaller chromatographic columns, and simplified chemical procedures is also expected to minimize procedural blank contributions and reduce isobaric interferences, thereby enhancing analytical reliability.

Despite these advantages, the technique has some limitations. In samples with very small grains and/or low apatite content, it can be challenging to obtain sufficient Sr material for isotopic analysis, especially in single grains, which can compromise the precision of the results in TIMS measurements. Figure 5 shows the behavior of analytical uncertainty and the number of apatite grains in the samples analyzed in this study. The presence of grains with complex internal structures and/or mineral inclusions [61, 62] not identified by binocular magnifying glass can also negatively affect the quality of the results. In addition, although the proposed method may be affected by secondary chemical or isotopic alterations in apatite or by the presence of inherited grains, these factors mainly influence the digestion efficiency of individual grains under the conditions used in this procedure. The presence of secondary alteration and/or inherited domains within apatite grains may affect the Sr isotopic composition obtained by the proposed method. Under the chemical digestion conditions employed in this study, altered domains or inherited components may be dissolved either partially or completely, depending on their chemical characteristics and dissolution behavior. Consequently, the Sr released during digestion may represent a mixture of Sr from different domains within the grain, each potentially having distinct Sr isotopic compositions. In such cases, the measured ^87^Sr/^86^Sr ratio may differ from the isotopic composition of the primary magmatic apatite or from that of the dominant domain within the grain.

**FIGURE 5:**
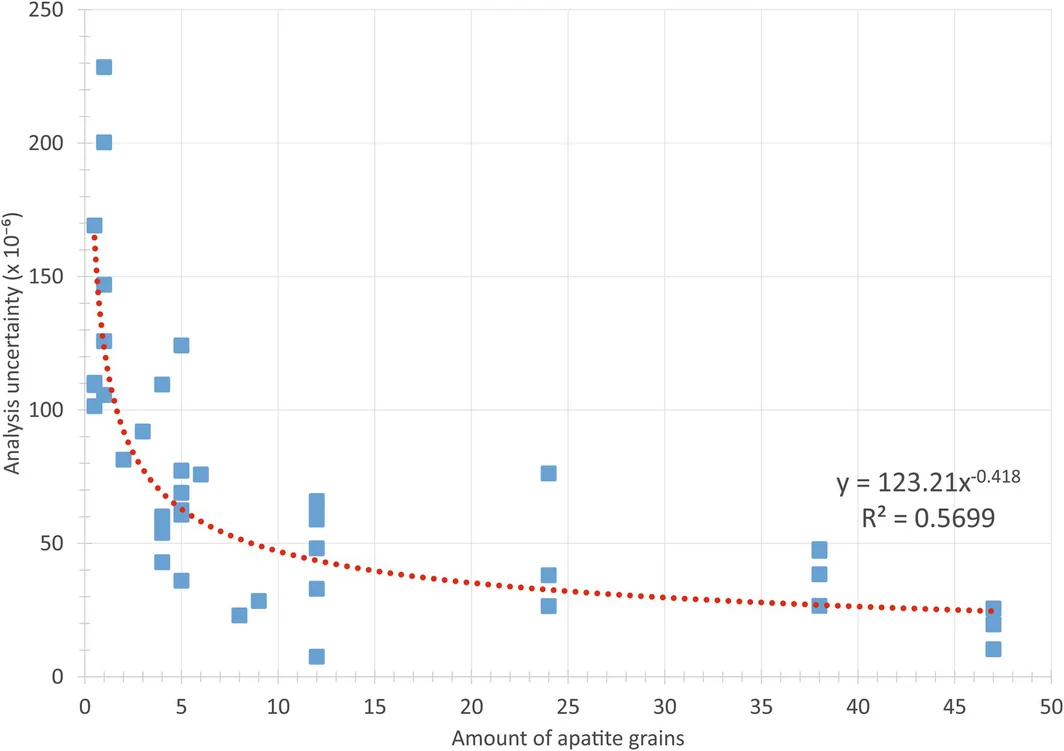
Analytical uncertainty of ^87^Sr/^86^Sr measurements (blue square) as a function of the number of apatite grains analyzed. The red dotted line represents the potential trend fitted to the data (*R* ^2^ = 0.5699).

## 5 Conclusions

The TIMS technique presented here combines chemical and spectrometric procedures for analyzing Sr isotopes in apatite grains, exhibiting low variability with repeated measurements of the same sample differing by less than 0.05%, and demonstrating precision and reproducibility. The developed method validates the use of Sr isotopic composition in apatite as a proxy of ^87^Sr/^86^Sr initial ratio in granitoid rocks. The feasibility of the technique is corroborated by the reproducibility obtained from reference materials, such as EN‐1 (USGS) and Durango Apatite.

The methodological optimizations implemented in this study demonstrate that precise and reproducible Sr isotopic measurements can be achieved in apatite grains with extremely low‐Sr contents, providing a robust and cost‐effective alternative to conventional whole‐rock analytical approaches. This approach stands out for its efficiency in reducing analytical cost and time compared to the technique reported by Neto et al. [36], while maintaining high analytical precision.

The initial whole‐rock ^87^Sr/^86^Sr ratios, calculated from crystallization ages, measured ^87^Sr/^86^Sr values, and Rb and Sr concentrations, exhibit an uncertainty of approximately 0.5% when compared with the average ^87^Sr/^86^Sr ratios obtained from apatite grains in representative pre‐, syn‐, and postcollisional granites of the Ribeira Belt. These results demonstrate the feasibility of obtaining ^87^Sr/^86^Sr ratios from apatite grains using TIMS and provide an initial assessment of the relationship between initial Sr isotopic compositions derived from apatite and those of the whole rock in their respective magmatic systems.

A critical aspect of the proposed procedure is its analytical performance under very low‐Sr loads, which represent one of the main challenges in TIMS isotopic measurements of apatite. In this study, the analytical performance was evaluated over a wide range of Sr masses (0.5–50 ng), reflecting the natural variability observed in apatite grains. Despite the low‐Sr amounts, the developed procedure produced reproducible isotopic data, with variability lower than 0.05% for both the reference materials and the studied samples.

Importantly, even analyses performed with sub‐ng to approximately 50‐ng Sr loads did not show systematic bias or significant analytical precision issues regarding the regional‐scale study proposed here. This behavior demonstrates that the optimized chemical separation procedures minimized the limitations commonly associated with low‐Sr TIMS analyses. The absence of significant isotopic fractionation related to Sr mass variations further reinforces the robustness of the analytical protocol.

Furthermore, the comparison between the ^87^Sr/^86^Sr ratios measured in apatite grains and the initial isotopic ratios calculated from whole‐rock data provides an assessment of the correspondence between apatite‐derived and whole‐rock Sr isotopic compositions in the investigated samples. Although differences were observed between the apatite and whole‐rock initial ratios, the results demonstrate the feasibility of obtaining Sr isotope compositions from low‐Sr apatite grains using the optimized TIMS procedure and provide an initial assessment of their relationship with the isotopic composition of the host granitoids.

Given the limited number of samples and their restriction to a single orogenic system, these results should not be considered evidence of the general applicability of apatite as a proxy for whole‐rock initial ^87^Sr/^86^Sr ratios. Further studies involving a larger number of samples from diverse geological settings will be required to evaluate the broader geological applicability of this approach.

## Author Contributions

**Carla Neto:** conceptualization, investigation, writing – original draft, methodology, validation, visualization, formal analysis. **Claudio de Morisson Valeriano:** conceptualization, funding acquisition, supervision, writing – review and editing, writing – original draft, resources, methodology, validation, investigation. **Gabriel Paravidini:** investigation, writing – review and editing, visualization, methodology, writing – original draft, formal analysis. **Henrique Bruno:** writing – review and editing, methodology, validation, funding acquisition. **Manuela Carvalho:** writing – review and editing, methodology, validation. **Rasec Almeida:** methodology, validation, writing – review and editing. **Caroline Peixoto:** visualization, software, data curation. **Samuel Bersan:** writing – review and editing, validation. **Mariana Carvalho:** visualization, software. **Monica Heilbron:** writing – review and editing, funding acquisition, resources.

## Funding

This work was supported by the Fundação Carlos Chagas Filho de Amparo à Pesquisa do Estado do Rio de Janeiro, Conselho Nacional de Desenvolvimento Científico e Tecnológico, FAPERJ Manutenção de Equipamento Multiusuários (E‐26‐210‐065/2021), FAPERJ Temático (E‐28/2021), CNPq‐PQ (310585/2021‐0), FAPERJ‐CNE (E‐26/200‐420/2023), and CNPq‐INCT GeoAtlântico Institute (405653/2022‐0).

## Supporting information


**Figure S1:** Thin‐section photographs under cross‐polarized light of major mineral assemblages of the (A) SMM‐CMM‐172, (B) BG‐200, (C) BG‐14B.
**Text S1:** Petrographic characteristics of the studied samples.
**Equation S1:** —Uncertainty propagation for initial ⁸⁷Sr/⁸⁶Sr calculations.
**Table S1:** —Chemical procedures for Sr elution with modifications from Charlier et al. [37].
**Figure S2:** Sr and Rb elution profiles obtained during calibration of the Sr‐Spec chromatographic separation using Durango Apatite.
**Table S2:** —Operating parameters for TIMS Sr isotopic measurement.
**Figure S3:** —Comparison between conventional whole‐rock analytical workflows (A) and the optimized apatite TIMS procedure presented in this study (B).
**Table S3:** —TIMS Sr isotope data from EN‐1 RM.
**Table S4:** —TIMS Sr isotope data from Durango Apatite RM.
**Table S5:** —EN‐1 RM data from the GeoReM database.
**Table S6:** —TIMS isotope data of apatite grains from granitoid rocks samples.
**Table S7:** —TIMS Sr isotope data for granitoid rock samples, with measured and calculated initial ^87^Sr/^86^Sr ratios with associated combined standard uncertainties.
**Table S8:** —Durango Apatite RM data from the GeoReM database.

## Data Availability

The data that supports the findings of this study are available in the supplementary material of this article.
